# Dietary Phenolic Compounds Exert Some of Their Health-Promoting Bioactivities by Targeting Liver X Receptor (LXR) and Retinoid X Receptor (RXR)

**DOI:** 10.3390/foods12234205

**Published:** 2023-11-22

**Authors:** J. Abraham Domínguez-Avila

**Affiliations:** CONAHCYT-Centro de Investigación en Alimentación y Desarrollo A. C., Carretera Gustavo Enrique Astiazaran Rosas No. 46, La Victoria, Hermosillo 83304, SO, Mexico; abrahamdominguez9@gmail.com

**Keywords:** nuclear receptors, gene expression, mRNA expression, diet, fruits and vegetables

## Abstract

Consuming foods of vegetable origin has been shown to exert multiple health-related effects, many of them attributed to their phenolic compounds. These molecules are known for being bioactive across multiple cells and organs, with documented changes in gene expression being commonly reported. Nuclear receptors are signal transducers capable of regulating gene expression in response to endogenous and/or exogenous ligands. Liver X receptor (LXR) and retinoid X receptor (RXR) are two important nuclear receptors that can be acted on by phenolic compounds, thereby modifying gene expression and potentially exerting numerous subsequent bioactivities. The present work summarizes recent evidence of the effects of the phenolic compounds that are exerted by targeting LXR and/or RXR. The data show that, when LXR is being targeted, changes in lipid metabolism are commonly observed, due to its ability to regulate genes relevant to this process. The effects vary widely when RXR is the target since it is involved in processes like cell proliferation, vitamin D metabolism, and multiple others by forming heterodimers with other transcription factors that regulate said processes. The evidence therefore shows that phenolic compounds can exert multiple bioactivities, with a mechanism of action based, at least in part, on their ability to modulate the cell at the molecular level by acting on nuclear receptors. The data point to a promising and novel area of study that links diet and health, although various unknowns justify further experimentation to reveal the precise way in which a given phenolic can interact with a nuclear receptor.

## 1. Introduction

Consuming a diet rich in foods of vegetable origin, such as fruits and vegetables, has been associated with numerous health benefits, including maintaining a healthy weight/amount of adipose tissue, controlling glycemia, normalizing the serum lipid profile, preventing certain types of cancer, among others [1]. Most of these benefits can be attributed to the different bioactive compounds that they contain, including phenolic compounds, which are ubiquitous in plant-derived foods. These molecules have been thoroughly investigated in silico, in vitro, and in vivo, in addition to being studied as part of dietary interventions and epidemiological studies in human beings, where the evidence suggests that they are highly bioactive [2,3,4,5]. For example, their anti-diabetic [6], anti-lipidemic [7], anti-cancer effects [8], among various others, have been described. Significant efforts have also been made to determine how these effects are exerted, in other words, what cellular, metabolic, or genomic processes are being targeted and how.

Nuclear receptors are multifunctional proteins with ligand-activated transcription factor activity, whose participation is nearly universal across a cell’s metabolism [9,10]. They can be considered sensors that determine the presence, concentration, and activity/effect of other molecules, including those of endogenous origin, nutrients, xenobiotics, among others, and initiate an appropriate response at the gene expression level. In general, once an appropriate ligand binds to a nuclear receptor, it will lead to conformational changes that induce or stabilize dimerization with another one to form an active dimeric structure; these conformational changes also weaken interactions with repressors in favor of activators of transcription. This dimer binds directly to specific DNA sequences and, when active, recruits the necessary proteins and enzymes to form the transcription complex, culminating in changes to the transcription of one or more genes. The mRNAs transcribed are translated into effector proteins, which will ultimately lead to an appropriate cellular response that, once fully activated, will maintain or return the cell to a state of homeostasis, according to the initial stimulus or ligand. For example, an increase in pro-oxidant molecules can be interpreted as the need to synthesize more endogenous antioxidant enzymes, whose activity will then counter the pro-oxidant aggression and return the cell to an appropriate redox balance. Thus, nuclear receptors function as signal-transducing elements between a particular stimulus and the appropriate cellular response required, and have even been considered promising targets to prevent or treat certain diseases [11,12].

Using nuclear receptors as molecular targets to improve health is a strategy that has been considered previously with significant success. For example, in the case of dyslipidemia, fibrates are a currently available class of pharmaceuticals, whose mechanism of action is based on targeting a nuclear receptor to improve the patient’s lipid profile [13]. The use of most pharmaceutical options unfortunately comes with various caveats, such as side effects that can be unpleasant at best or can compromise the patient’s health at worst. This has made the search for safe molecules with similar potential a significant priority to maintain health; phenolic compounds could be considered for this purpose, but their efficacy, safety, and mechanisms of action must first be thoroughly investigated.

Liver X receptor (LXR) and retinoid X receptor (RXR) are two important nuclear receptors central to lipid and energy metabolism, among other pathways. Because of their roles in said processes, the interactions of certain bioactives (such as phenolic compounds) with them could be an important link that explains how consuming plant-derived foods leads to many documented health effects throughout the organism. The effects of phenolic compounds on nuclear receptors are also less reported, as compared to their actions on some effector proteins (such as specific enzymes) or on a physiological effect (such as changes in the lipid profile), while the specificity of a compound for a particular receptor isoform is an even less reported and understood phenomenon, further justifying the relevance of studying these interactions. Phenolic compounds are present in foods of vegetable origin, and have been explored as potential nuclear-receptor-stimulating agents that can induce a positive effect on the consumer; because of their safety and presence in food, this could be a worthwhile endeavor [14].

Before discussing the effects of phenolic compounds, the following sections will provide detailed information about the two nuclear receptors considered in the present work.

## 2. Liver X Receptor (LXR)

Two LXR isoforms have been described, LXRα (NR1H3) and LXRβ (NR1H2). In humans, mature LXRα consists of 447 residues and an approximate mass of 50 kDa; its expression is significant in the liver, with the kidneys, intestine, adipose tissue, and macrophages also expressing it. LXRβ is of similar length (460 residues) and mass (approximately 51 kDa), and is expressed ubiquitously [15,16,17]. Their structure is similar to most other nuclear receptors, and consists of a variable N-terminal domain, a conserved zinc finger DNA-binding domain, a hinge region, a ligand-binding domain, and a variable C-terminal domain [16,17]. One of their main roles is to regulate cholesterol and lipid metabolism, which makes them ideal targets to prevent or improve dyslipidemia. They form heterodimers with retinoid X receptor (RXR), which have a DNA-binding ability to allow the regulation of relevant genes at the transcriptional level. Their main endogenous ligands are oxysterols, which induce the formation of the LXR/RXR heterodimer, the active form that directly regulates the transcription of key genes related to reverse cholesterol transport, with the aim of normalizing or maintaining cholesterol and lipids in general within the homeostatic range. Additional evidence shows that LXRs are also involved in establishing an adequate immune response, regulating inflammation, and other important metabolic processes, further increasing their significance for health. In addition to their natural ligands, synthetic ones have also been developed and, of particular relevance to the present work, phenolic compounds of dietary origin could also interact with them [18,19,20,21,22,23].

## 3. Retinoid X Receptor (RXR)

Three RXR isoforms are known, RXRα (NR2B1), RXRβ (NR2B2), and RXRγ (NR2B3). In humans, the mature proteins consist of 462, 533, and 463 residues, respectively, with molecular masses of approximately 51, 57, and 51 kDa, respectively [16,24]. Their structure is similar to that of LXR and other nuclear receptors, with an N-terminal domain, a conserved zinc finger DNA-binding domain, a hinge region, a ligand-binding domain, and a variable C-terminal domain. Their expression varies across cell types and differentiation status but, in general, RXRα is expressed in the livers, kidneys, and intestine, RXRβ is ubiquitous, and RXRγ is expressed in the brain and muscle [24]. 9-*cis*-retinoic acid is their main ligand [25], while their role is remarkably varied and widespread across a number of metabolic functions, according to their ability to form heterodimers with LXR to regulate lipid metabolism (as previously mentioned), in addition to similar interactions that take place with the farnesoid X receptor (FXR), peroxisome proliferator-activated receptor (PPAR), pregnane X receptor (PXR), retinoic acid receptor (RAR, whose ligand is also 9-*cis*-retinoic acid, in addition to *all*-*trans*-retinoic acid), thyroid hormone receptor (TR), and vitamin D_3_ receptor (VDR). Moreover, it can also form homodimers that are similarly capable of regulating gene expression. RXRs permit binding to the hexameric consensus sequence 5′-AGGTCA-3′ that can be found in various response elements, such as the liver X response element (LXRE), among others. Specificity and precise regulation are provided by the directionality of repeats of this sequence (for example, direct, inverted, everted, or palindromic), and the number of nucleotide spacers between them [26]. As evidenced by the contrasting nature and number of binding partners of LXR, it is apparent that it can respond to multiple stimuli (at least indirectly) and participate in various metabolic processes. This makes it particularly interesting as a potential target since interacting with this single molecule could theoretically result in many effects across multiple cells/organs, such as those documented for phenolic compounds.

**Figure 1:**
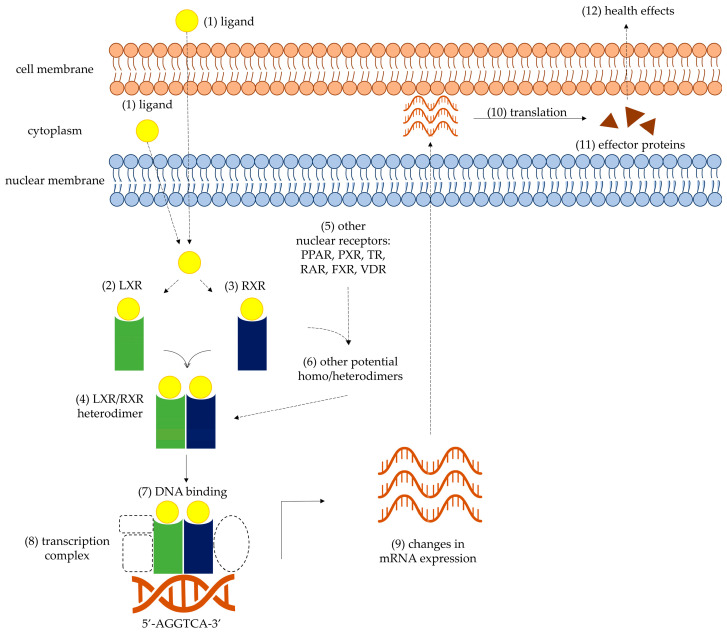
Overall process of LXR and RXR activation. An appropriate extra- or intracellular ligand (1, yellow circle), oxysterols for LXR (represented in green) or 9-*cis*-retinoic acid for RXR (represented in blue), will bind to its corresponding receptor (2, 3) and form an active LXR/RXR heterodimer (4), while also weakening interactions with repressors (not shown). It is also possible for RXR to dimerize with itself or with other nuclear receptors (5) and form additional potential homo- or heterodimers (6). The active LXR/RXR heterodimer can bind to the consensus sequence 5′-AGGTCA-3′ (7), which results in activation of the transcription complex (8, dashed figures) and changes in the mRNA expression pattern of the cell (9). The transcripts are translated (10) into effector proteins (11) that can then exert specific health effects (12), such as normalization of cholesterol/lipid metabolism or inflammatory processes.

**Figure 2:**
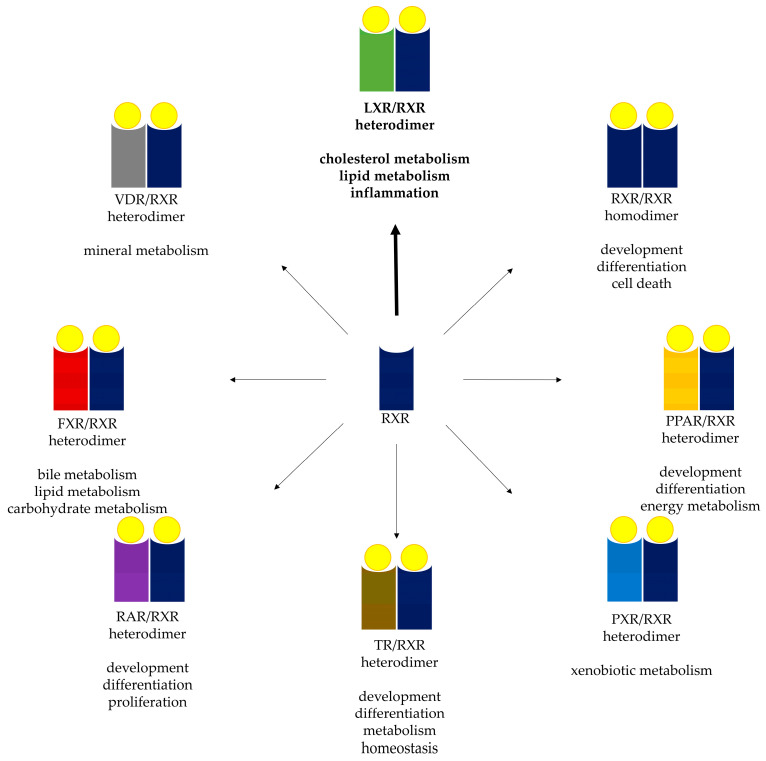
Overview of the metabolic processes modulated by RXR (center, represented in blue) and other nuclear receptors (represented in a different color for each one) with which it dimerizes. RXR can interact and dimerize with other nuclear receptors; clockwise from top-center, the LXR/RXR heterodimer mainly regulates cholesterol/lipid metabolism and inflammation. Thus, it has been widely associated with maintaining or normalizing serum cholesterol and the lipid profile overall. RXR can also dimerize with itself and modulate basic developmental and differentiation processes. The PPAR/RXR heterodimer regulates overall energy metabolism, as well as cellular differentiation. The PXR/RXR heterodimer responds to xenobiotic compounds, leading to the transcription of the genes necessary to prevent their accumulation and potential cell damage. The TR/RXR heterodimer responds to thyroid hormones, thereby participating in basal processes across most cells. The RAR/RXR heterodimer responds to retinoic acid isomers (9-*cis* or *all*-*trans*) and is associated with various basal metabolic, proliferation, and homeostatic processes. The FXR/RXR heterodimer responds to bile acids, and promotes the transcription of genes associated with their metabolism. Finally, the VDR/RXR heterodimer responds to vitamin D_3_ (calcitriol), the active form of this vitamin, thereby participating in maintaining calcium and other mineral homeostasis. Because of the multiple processes under the direct or indirect control of RXR, changes in its activity by dietary phenolic compounds can result in numerous bioactivities across multiple cells and organs.

Figure 1 depicts the overall process of activating LXR and RXR, while Figure 2 summarizes the overall metabolic processes modulated by RXR and other nuclear receptors with which it dimerizes.

The potential of nuclear receptors as molecular targets has been previously recognized: for example, Hiebl et al. [27] reviewed the effects of natural products on LXR, FXR, and RXR. However, they considered multiple types of bioactive compounds. The present work focuses on the effects of phenolics from multiple sources and across different experimental models, as discussed in the following section, in order to reveal their health-associated bioactivities, whose mechanisms of action are related to targeting LXR and/or RXR. It should be noted that 48 nuclear receptors are currently known, although the metabolic roles of LXR and RXR make them particularly well suited to respond to dietary compounds, and their effects can impact numerous cellular processes. Moreover, the effects of phenolic compounds on another nuclear receptor, PPAR, have already been described [28], which suggests that targeting nuclear receptors may be a common way in which phenolic compounds are able to exert some of their bioactivities.

## 4. Bibliographic Analysis

The Web of Science database was used to search for high-quality peer-reviewed documents. The terms “phenolic compounds”, “polyphenols”, and “phenolics” were used, alongside “liver X receptor” or “LXR” and “retinoid X receptor” or “RXR”, limited to the 2013–2023 period. Only original papers were included (reviews were not considered). The main criterion used to include an article in the present work was if it reported an experimental analysis of LXR/RXR in response to phenolic compounds or a sample that contained them; 129 total articles were found, of which 67 met said criterion. From these, approximately half were chosen to discuss in detail, in order to illustrate as broadly as possible the effects of different phenolic compounds, vegetable sources, experimental models (in silico, in vitro, and in vivo), synthetic/derivative compounds, and combining different compounds.

## 5. Effects of Various Phenolic Compounds on LXR and RXR

### 5.1. Quercetin

Dietary interventions have shown that phenolic compounds are highly bioactive and health-promoting, with some of their mechanisms of action being directly or indirectly related to LXR, according to various forms of experimental evidence. For example, Lu and Jia [29] used THP-1 (human leukemia monocytic) cells, that were differentiated into foam cells (cholesterol-rich macrophages that are known to be atherosclerotic plaque-forming cells in vivo) using oxidized LDL particles, with three quercetin concentrations as treatment (5, 10, or 15 μM). Real-time PCR and Western blot analyses evidenced a significant effect on ATP-binding cassette transporter 1 (ABCA1), a cholesterol efflux protein that promotes reverse cholesterol transport. Moreover, it is noteworthy that the effects were detected at both the mRNA and protein level, and that changes were dose-dependent. Changes in the expression of this transporter were correlated with a similar dose-dependent increase in LXRα, suggesting that quercetin targeted this nuclear factor, which then induced ABCA1 transcription in order to normalize the lipid profile via promoting reverse cholesterol transport. This was further confirmed by additional experimental evidence where, after transfecting cells with LXRα small interfering RNA (siRNA), the quercetin-induced effect was not apparent anymore. The authors propose that these findings support the role of quercetin as an antiatherogenic agent, according to its potential to induce cholesterol efflux from lipid-rich, plaque-forming cells, via a mechanism of action that involves targeting LXRα. Since the normal physiological function of LXRs is to modulate lipid metabolism, it is apparent that quercetin may stimulate them and potentiate this bioaction.

Waizenegger et al. [30] performed an in vitro transcriptomic study using quercetin. They first used an ingenuity pathway analysis, which predicted that the FXR/RXR and PXR/RXR pathways would be activated via hepatocyte nuclear factor 4α (HNF4α) signaling, another nuclear factor that can regulate RXR. In contrast to this predicted result, experimental data on HepG2 (human hepatocellular carcinoma) cells revealed that HNF4α and some of its target genes (including RXR) were instead downregulated, which led the authors to propose an inhibitory effect of quercetin on HNF4α-mediated signaling and, therefore, on RXR. Additional experimental data on HEK293 (human embryonic kidney) cells also revealed that, although quercetin decreased the expression of other receptors like FXR, LXRα, and PXR (all of which form heterodimers with RXR), the expression of RXRα itself did in fact increase. This apparently conflicting information made the authors propose that quercetin exerts a “regulatory role” on the organism, and that its effects are highly dependent on dose. Of note, changes at the protein level could have taken place since all of the aforementioned data are for mRNA expression. Moreover, since RXR responded to the treatment, but not its obligate heterodimer-forming nuclear factors, it is possible that some of the mechanisms associated with, for example, FXR, LXR, or PXR are not exerted directly on them, but instead target RXR and exert some actions through it; however, this cannot be confirmed nor denied using the results described.

### 5.2. Resveratrol

Pang et al. [31] fed male C57BL/6J mice high-fat only or high-fat, cholesterol, and bile salt diets in order to induce hypercholesterolemia. The animals were treated with resveratrol, either via oral gavage (0.8–500 mg/kg body weight) or supplemented in their diets (0.1 or 0.5% *w*/*w*). The authors report significant improvements to the animals’ serum cholesterol, triacylglycerols, LDL, insulin, and glucose, as well as to their hepatic cholesterol and triacylglycerols. The effects were shown to be dose-dependent, and were also reflected in improved tissue markers, as evidenced by the histological non-alcoholic fatty liver disease (NAFLD) activity score scale. An increased excretion of fecal sterols prompted further in vitro experiments on the Caco2 (human colorectal adenocarcinoma) cells, which revealed that resveratrol was able to induce the activation of LXRα (but not LXRβ) and cholesterol transporters (ABCB1, ABCG5, and ABCG8), thereby explaining the sterol-excreting effect documented in vivo. If LXRα was knocked down, the resveratrol-induced effects on the cholesterol transporters were significantly inhibited, further confirming that resveratrol was in fact exerting its bioactivities via LXRα. Because resveratrol appears to be selective to LXRα, some specific interactions could be occurring; however, this was not explicitly studied, suggesting the need to determine the actual phenolic-protein interactions that take place between resveratrol and LXRα, but not with LXRβ. If selective activation can be confirmed using additional analyses, targeted derivatives could be considered to selectively alter specific metabolic pathways without interfering with others.

The use of resveratrol was explored by Maj et al. [32] as an antiproliferative agent in vitro. The authors treated different malignant cells lines for 72 h with this compound (1, 25, 125, or 625 μM), or with a vitamin D analog [(24R)-1,24-dihydroxycholecalciferol, PRI-2191] (1, 10, 100, or 1000 nM). The same resveratrol doses were also co-administered with 100 nM PRI-2191. Western blot analyses showed that resveratrol upregulated the expression of RXRα in some cell lines, but downregulated it in others. When it was administered in combination with PRI-2191, it was able to improve its effects on VDR/RXRα target genes (CYP24A1), suggesting a potential synergy between these compounds. It is also interesting that changes at the mRNA expression level were not detected, which could indicate functional effects that may not depend on enhanced transcription, but instead take place as phenolic–protein interactions. Because the changes were not consistent across cell lines, the authors propose that resveratrol-mediated effects were cell-specific; thus, the effects of this compound on RXR may only be apparent in some cells/tissues or even only in some organisms. It is also noteworthy that the cell lines studied had different mutations and epigenetic profiles, which may further complicate how they respond to certain compounds; nevertheless, the effects observed suggest the at least partial effects of resveratrol on RXRα at the protein level. The effects of related analogs (PRI-1916 and 1917) combined with carnosic acid have also been shown to mediate VDR/RXR signaling in vitro [33], suggesting the interesting potential of such combinations.

Stone et al. [34] determined the potential of resveratrol to act as a vitamin D receptor-signaling modulator. Reporter vectors were constructed based on various response element sequences, which were then transfected into Caco-2, HEK293, and C2C12 (mouse myoblast) cell lines, which were then treated with resveratrol (10^−5^ M) and/or 1,25-dihydroxyvitamin D_3_ (10^−8^–10^−10^ M). The results showed that resveratrol was able to stimulate vitamin D receptor transcription, which was due to its ability to promote the formation of the VDR/RXR heterodimer. Interestingly, point mutation assays showed that resveratrol can act as an allosteric modulator that increases the formation of the VDR/RXR heterodimer and/or stabilizes it. Evidence on other RXR heterodimers suggested that resveratrol preferentially targeted RXR (instead of its binding partner), which resulted in increased heterodimerization, the subsequent formation of the transcription complex, and changes in gene expression. Thus, resveratrol could simultaneously have an impact on multiple cellular processes by acting on RXR-mediated signaling pathways; its effects on health could be wide-reaching due to this particular mechanism of action. In contrast to the aforementioned results, Escolà-Gil et al. [35] report that resveratrol does not have an effect on LXR signaling or reverse cholesterol transport in vivo (C57BL/6 mice). Conversely, Choi et al. [36] argue that resveratrol and a synthetic derivative (SY-102) can exert hepatoprotection in ICR mice, by acting on the AMPK/LXR/SREBP pathway. Lee et al. [37] also used a resveratrol derivative, oxyresveratrol, and showed its significant bioactivity, according to its potential to inhibit hepatic lipogenesis and therefore exert hepatoprotective effects, by regulating the LXRα/SREBP-1c pathway in C57BL/6 mice fed high-fat diets (HFDs). These contrasting findings suggest that the effects of resveratrol and its derivatives may be dependent on the specific model used.

### 5.3. Chlorogenic Acid

Huang et al. [38] studied chlorogenic acid (5-caffeoylquinic acid), a compound characteristic of coffee and other foods and beverages of vegetable origin. Male Sprague-Dawley rats were fed an HFD and treated (oral gavage) with a low (20 mg/kg) or high (90 mg/kg) chlorogenic acid dose once daily. After a 12-week period, the results showed an HFD-induced increase in hepatic LXRα mRNA expression; however, the treatment decreased it to normal values, similar to those of animals fed a standard diet. The expression of RXRα increased only in response to the high dose (and only as compared to the HFD group), but not in the other groups. Based on these results and on the additional hypolipidemic and hepatoprotective effects documented, the authors proposed that an activation of hepatic LXRα would result in hypertriglyceridemia and hepatic steatosis via sterol regulatory element-binding protein 1-c (SREBP-1c, an important regulator of lipid metabolism); thus, maintaining it within normal values (as was found in response to the treatment) is crucial to preserve liver health. Regarding RXRα expression, the authors argue that it was associated with increased fatty acid oxidation via the PPARα/RXRα heterodimer since the treatment also stimulated it. The overall data reported in this study suggest that the effect of a phenolic compound is not limited to stimulating a nuclear receptor, but it may also prevent an increase and maintain normal values under certain circumstances. Moreover, since proteins were not specifically studied, only mRNA expression, complementary effects could have also occurred at the protein level, which would provide additional fine-tuning to the anti-obesity and tissue-protective bioactivities described.

Yin et al. [39] used a largemouth bass (*Micropterus salmoides*) model fed an HFD, supplemented with two doses of chlorogenic acid (300 or 600 mg/kg diet) during a nine-week period. The phenolic treatments induced various improvements to the lipid profile, in addition to antioxidant and anti-inflammatory effects. The LXRα expression decreased in response to the HFD, although the low-dose treatment was able to increase it to normal values. No changes were recorded in RXRα, while a decrease was documented for FXR, which the treatments were not able to counter. Based on these findings, the authors proposed that LXR was sensitive to the chlorogenic acid treatments, which resulted in maintaining cholesterol homeostasis. Changes in bile metabolism in response to the treatments were interpreted as the compound having the ability to modulate it, although this was apparently not exerted via the classical pathway, according to the expression of FXR and other bile-related genes. It is also possible that changes in RXR, FXR, and/or additional genes could have been exerted at the protein level, since these data are only for mRNA expression; however, this cannot be determined with the present results.

Due to the presence of chlorogenic acid in coffee, Xu et al. [40] investigated the combined effect of this compound with caffeine as an anti-obesity treatment. Female ICR mice were fed an HFD supplemented with chlorogenic acid (0.2%), caffeine (0.04%), or both compounds combined (chlorogenic acid 0.1 or 0.2% and caffeine 0.02 or 0.04%). After a 14-week period, their weight increased in response to the diet, as did the lipid profile, both of which the chlorogenic acid/caffeine combination were able to mitigate. Although some effects were found with individual compounds, caffeine in particular, the combination exerted the strongest anti-obesity actions. The mRNA expression of LXRα increased in response to the HFD, which the combined treatment was able to mitigate, although it still remained higher than the control fed a standard diet. Regardless, the modification was sufficient to induce the aforementioned changes, which the authors attributed to the upstream effects on phosphorylated AMP-activated protein kinase (AMPK), an “energy sensor” that regulates various metabolic processes via LXRα (among others). Although chlorogenic acid by itself was not able to induce significant changes (as compared to its effects when combined with caffeine), the results documented highlight a synergy between the two compounds studied. This is particularly relevant because chlorogenic acid and caffeine are co-consumed in coffee; thus, at least some of the reported effects of this beverage on lipid/energy metabolism could be attributed to the phenolic–caffeine synergy described, which acts via the AMPK/LXRα pathway. The authors emphasize this by commenting that the administered doses are comparable to what is consumed daily in regular coffee. In contrast to these findings, the related compound 3-caffeoyl, 4-dihydrocaffeoylquinic acid was administered to HepG2 cells treated with high glucose concentrations, and was found to not exert a normalization of mRNA expression, which suggests that the chemical structure of caffeoylquinic acids is important to exert their modulating effects on LXRα and other nuclear receptors [41], in addition to potential synergies with other compounds, as previously stated.

### 5.4. Cyanidin

The effects of cyanidin were studied by Jia et al. [42]. The authors used CHO-K1 (Chinese hamster ovary) cells and a luciferase reporter assay to determine whether cyanidin (5, 10, 50, or 100 μM) would induce LXR. An increase was apparent in LXRα in response to 50 or 100 μM cyanidin, while only 100 μM resulted in the same effect on LXRβ, which suggests a slight preference for the former. Further experiments confirmed a higher affinity of cyanidin for LXRα, according to a dissociation constant (K_D_) of 2.16 μM and half-maximal effective concentration (EC_50_) of 3.48 μM for this isoform, as compared to a K_D_ of 73.2 μM and EC_50_ of 125.2 μM for LXRβ. The authors comment that the recorded affinity of cyanidin is similar to that of LXR’s natural ligands (oxysterols), since the most potent one has an EC_50_ of approximately 1 μM; thus, potential competition could occur between them. The in vitro data showed that the interactions of cyanidin with LXR were able to increase the mRNA expression of genes regulated by LXRα, but not by LXRβ, once again providing support for its contrasting affinity for each isoform. Moreover, cyanidin was able to decrease the lipid content of macrophages and hepatocytes via its LXR-related effects. These data provide additional evidence for the significant effects of phenolic compounds on LXR, while also demonstrating how phenolic–protein interactions can vary from one isoform to another, according to the higher affinity for the α isoform, as compared to β.

The glycoside (cyanidin-3-O-β-glucoside chloride) and aglycone (cyanidin chloride) forms of cyanidin have both been shown to modulate LXRα. Du et al. [43] used HK-2 (human kidney) cells that were pre-treated with either compound (50 μM for 1 h), before subjecting them to a high glucose concentration (30 mM for 24 or 48 h), as an in vitro model of diabetic nephropathy. The results showed a potential cholesterol efflux induced by the treatments, which was attributed to the ABCA1 transporter. Further experimentation revealed that this action was partly modulated by LXRα, since silencing its expression resulted in decreased ABCA1 expression and its associated effects on cholesterol efflux. In addition to the lipid-associated effects, the anthocyanins considered also exerted anti-inflammatory actions, according to decreased cytokine production. Modulating LXRα was thus also shown to be a mechanism by which the anti-inflammatory effects were exerted. It should be noted that the effects were also dependent on PPARα and NFκB, not exclusively on LXRα. These data therefore suggest that anthocyanins are capable of simultaneously exerting effects on the lipid and inflammatory metabolism of pretreated HK-2 cells, both of which are due to modulating the LXRα pathway, in addition to associated ones.

### 5.5. Mixed Phenolics

Little et al. [44] administered quercetin and ferulic acid (1 μM of each or both) to human adipocytes cultured under conditions that the authors describe as an “ongoing lipogenic state” and a “lipid storage stage”, which intended to mimic cells that were actively synthesizing and accumulating lipids and cells that were only maintaining them, respectively. They determined that the treatments exerted different results, both on the effects themselves (lipid concentration and profile) and on the genes involved. For example, on the “lipid storage state”, qualitative and quantitative changes in the lipid profile were documented, which were determined to be under the control of the PPARα/RXRα pathway. In contrast to this finding, cells that were at the “ongoing lipogenic stage” were apparently not related to PPARα/RXRα. Their results allowed the authors to propose that cells responded better to the phenolic treatments when they were in a basal state, i.e., not actively synthesizing lipids, according to increased lipolysis and a decreased lipid content, both of which were apparently mediated via PPARα/RXRα. This was further interpreted to propose that consuming these compounds in the diet may decrease lipid accumulation and the browning of adipose tissue, in favor of a more lipolytic phenotype. The notion of “prophylactic” instead of “therapeutic” actions of bioactive compounds has been considered before, and supports the idea that an adequate diet may be more effective when consumed before a disease is established [45], as can be suggested from these results.

Fouache et al. [46] selected four flavonoids (galangin, quercetin, apigenin, and naringenin), which they used to perform in vitro and in silico analyses to determine their effectiveness as LXR modulators. HeLa (human cervical cancer) cells were transfected with a reporter vector and stimulated with up to 100 μM of each individual compound. They were able to demonstrate that quercetin stimulated both LXRα and LXRβ, and apigenin stimulated LXRβ only, while galangin and naringenin were unable to show any activity on either isoform. Subsequent analyses revealed the effect of said flavonoids on known LXR-regulated genes, specifically ABCA1, SREBP-1c, and fatty acid synthase (FASN). These results showed an increase in ABCA1 exerted by quercetin and apigenin, no effect on SREBP-1c by any compound, and increases in FASN exerted by apigenin, galangin, and naringenin (all except quercetin). It should be noted that said effects differed when the flavonoid was co-administered with T0901317, a known LXR agonist, suggesting that these compounds are sensitive to the presence of other molecules. Even while the compounds studied have similar chemical structures, the in silico data revealed that subtle differences from one flavonoid to another can result in drastically modified activities, such as changing from acting as an agonist to an antagonist, while they can also alter their affinity from one isoform to another, both, or neither. This is due to, as the authors determined in silico, a number of interactions that take place in specific binding pockets, where each compound can form hydrophobic interactions and/or hydrogen bonds, which leads to the aforementioned changes in LXR.

Zhang et al. [47] studied the effect of an activated charcoal sorption of a Chinese herb extract (*Pulsatilla chinensis*, *Portulaca oleracea* L., *Artemisia argyi Folium*, and *Pteris multifida Poir*) rich in phenolic acids and flavonoids, that was administered as part of the diet of broilers (250 mg/kg diet). Cultivated chicken hepatocytes were also treated to determine changes in gene expression in response to the treatment (200 μg/mL), as well as the main phenolic acids (caffeic acid and vanillin, 150 μg/mL) and flavonoids (daidzein and quercetin-d-glucoside, 50 μg/mL) that it contained. The results showed that phenolic acids by themselves or combined with flavonoids (but not flavonoids by themselves) increased the mRNA expression of RXRα. Moreover, flavonoids and phenolic acids combined with flavonoids were able to increase the mRNA expression of PXRα, CYP7A1, and other genes related to hepatic bile acid and xenobiotic metabolism. According to these results, both RXR and PXR responded to phenolic acids, flavonoids, or both combined, suggesting an effect on bile and xenobiotic metabolism. Indeed, an increase was documented in gallbladder and fecal bile acids in the treated animals, which was interpreted as an enhanced fecal excretion of bile acids, that was consequent to the treatment administered. The data apparently suggested that the effects of the herb extract could be attributed to its phenolics as major regulators of RXR and PXR which, in the liver, activate bile and xenobiotic metabolism. It can also be argued that phenolic compounds were most effective when combined since the magnitude of individual treatments (phenolic acids or flavonoids) was lower than that of their combination. This points to a significant synergy between phenolics, as demonstrated in numerous studies, and which more closely mimics the potential effects of consuming them normally, since a regular everyday diet will include various phenolics and numerous additional compounds, not just some of them in isolation.

Curcumin is the main and most studied phenolic contained in turmeric (*Curcuma longa*); it has shown significant bioactivity regarding its potential to modulate RXR or LXR, for example, in cancer stem cells [48], in rats with cerebral hypoperfusion [49], and in rats fed high-fat high-fructose diets [50], as well as various others unrelated effects. Such bioactivities have led to an increased interest in its study: for example, Batie et al. [51] developed synthetic halogenated derivatives of the parent molecule, and analyzed them in combination with resveratrol. Luciferase and mammalian two-hybrid (M2H) assays were used to determine whether VDR/RXR heterodimerization and changes in gene expression occurred in response to the compounds administered (3.75 × 10^−5^ M). The authors determined that curcumin and some of the derivatives tested were able to induce signaling, thereby proposing that the modifications made to the original chemical structure retained its ability to modulate VDR/RXR-mediated signaling. Moreover, when resveratrol was simultaneously added (2.8 × 10^−5^ M) with either curcumin, its derivatives, or the active form of vitamin D_3_ (the physiologic ligand), the effects increased significantly, suggesting a potential synergy between resveratrol and curcumin or the physiologic ligand.

### 5.6. Phenolic Extracts

Jimenez-Aspee et al. [52] prepared a *Prumnopitys andina* fruit extract and identified a series of phenolic compounds, including derivatives of hydroxycinnamic acids and flavonoids like shikimic acid, protocatechuic acid, caffeic acid, ferulic acid, apigenin, isorhamnetin, and quercetin (among others). These extracts were then used to determine whether they would affect the transcription of LXRα or LXRβ in HepG2 cells. As hypothesized, when treating them with 50, 100, and 200 μg/mL (but not 1 or 10 μg/mL), a significant increase was found in both receptors. The authors attribute their findings, at least in part, to apigenin, due to its resistance to the digestive process and to previous reports; however, synergy between compounds cannot be ruled out when administering a complex mixture like the one used in this experiment. The authors then determined whether their extract would change the expression of genes under the transcriptional control of LXR, and report that ABCA1, SREBP-1c, and FAS did increase their mRNA expression in response to the phenolic-rich extract. When a recorded effect is due to the administration of a complex mixture, such as the extract used in this work, it can be difficult to determine precisely which compound was responsible for it; however, it should be noted that a normal diet is not made up of pure isolated compounds but a combination of numerous molecules where varied interactions between them take place. Recording a bioactivity exerted from the intake of such a matrix remains relevant, even if it is impossible to determine which compound was responsible for it.

Danielewski et al. [53] prepared a Cornelian cherry (*Cornus mas* L.) extract, which was purified using amberlite resin to concentrate iridoids (monoterpenoids) and phenolic compounds, particularly anthocyanins, phenolic acids, and flavonols. The extract was administered (10 or 50 mg/kg bw, oral gavage, single daily dose) to male New Zealand rabbits that were fed a 1% cholesterol diet. The authors performed Western blot analyses to determine the hepatic protein expression of LXRα after a 60-day intervention, and found that both treatments increased it, particularly the 10 mg/kg bw dose. Although the mRNA or protein expression of LXR-controlled genes was not determined, the lipid profile did show improvements in response to the treatments, suggesting that some processes like reverse cholesterol transport may have been favorably altered. This experiment also reveals the need to determine an effective dose since the strongest response was exerted by the lower concentration, highlighting that a higher dose is not always better when it comes to consuming a given treatment. Interestingly, others report that non-anthocyanin phenolics from cherries (*Prunus avium* L.) are also capable of exerting hepatoprotective effects in obese diabetic (db/db) mice by acting on LXRα [54], further supporting the potential of cherry phenolics to act on this transcription factor.

Mokhtari et al. [55] used loquat (*Eriobotrya japonica* (Thunb.) Lindl.) fruit peels as a source of phenolics, due to evidence of the ethnopharmacological potential of the fruit. A hot water extraction method was used to obtain an extract, which was then administered to male albino mice (100 or 200 mg/kg/day, oral gavage), that were fed a high-fat high fructose diet. After a 45-day period, various abnormalities were documented in the lipid profile, as expected after consuming a simple-carbohydrate- and fat-based diet. The extract showed dose- and time-dependent effects on the lipid profile, which can be summarized as a decreased atherogenic index. In addition, hepatoprotective and antioxidant effects were documented, which further supported the health-promoting effects of the treatment. The authors performed additional in silico analyses to determine the potential interactions that could have been responsible for the observed effects. They analyzed the interactions of six compounds or their metabolites present in the extract administered (caffeoylquinic acid, chlorogenic acid, ferulic acid-4′-O-sulfate, caffeic acid-4′-sulfate, quercetin-3-O-glucuronide, and quercetin-3′-O-sulfate) with various potential targets (LXRα, RXRα, RXRβ, RXRγ, and others). Of relevance to the present work, it was found that all six metabolites could theoretically interact with LXRα, RXRα, and RXRγ, while four of them could interact with RXRβ. This information allowed the authors to propose that the loquat treatments administered could be exerting their anti-lipidemic effects via LXR-mediated reverse cholesterol transport. Other actions could also be happening via interactions with FXR, PPAR, or other effector proteins. Altogether, this study provides evidence that the possible mechanisms of action of phenolic compounds could be related to their interactions with nuclear receptors and, of notable relevance, their biotransformed metabolites (sulfated or glucuronidated) were also evaluated. The authors’ consideration of said metabolites is noteworthy since phenolic compounds are subject to significant first-pass metabolism that leads to the production of these molecules; thus, the parent compound and/or its metabolites may be responsible for the documented effects.

Catechins are characteristically found in cocoa and green tea, among other foods and beverages. Oleaga et al. [56] propose that cocoa flavonols (mainly epicatechin and phenylacetic derivatives, and vanillic acid) modulate HDL and apolipoprotein A1 mRNA, according to RXRα electrophoretic mobility shift assays. Green tea catechins were shown by Hao et al. [57] to increase the mRNA expression of all three RXR isoforms (α, β, and γ) in a rat model (F344 rats) of colorectal cancer (azoxymethane-induced), which resulted in anti-tumorigenic effects. The highly bioactive epigallocatechin-3-gallate (EGCG) has also been shown to act as an anticarcinogenic compound by Morris et al. [58] in various CpG island methylator phenotype + (CIMP+) cell lines (where genes are epigenetically silenced, as occurs in certain malignancies), according to its ability to restore the expression of silenced RXRα, potentially indicating its anti-cancer effect. Taken together, these experiments suggest that different catechins and sources that contain them exert effects that positively impact both cardiovascular health (via HDL and lipoproteins) and carcinogenic processes (via normalized gene expression) by acting on RXR isoforms.

Li et al. [59] prepared an apple polyphenol extract, and administered it (125 or 500 mg/kg/day) to C57BL/6 male mice that were fed an HFD (60% fat) for 12 weeks. Various health effects were documented in response to the treatment: for example, the HFD decreased the diversity of the gut microbiota, which the apple phenolics were able to counter. The liver was also likely protected, according to an increased cholesterol/bile efflux that acted against diet-induced hepatic steatosis. Regarding LXR, its ileal expression increased in response to the higher dose of apple phenolics, a change that was potentially responsible for a concomitant modification to the expression of cholesterol-efflux-related genes. These results suggest the direct hepatic and intestinal protective effects of apple phenolics, although their impact on health can extend beyond these organs to the organism as a whole.

Domínguez-Avila et al. [60] administered pecan nuts (*Carya illinoinensis*) to Wistar rats fed an HFD, in addition to also separately administering its oil or its phenolics (0.1% *w*/*w*) as part of their diets. After a nine-week period, it was found that pecan and its fractions countered various effects of the HFD: among them, pecan phenolics were able to significantly increase the mRNA expression of hepatic LXRα. It is noteworthy that the effect of phenolics was significantly higher than that exerted by either pecan oil or whole pecans, suggesting a clear targeted effect, which appeared to have exerted a potential anti-atherogenic effect. However, this increase in LXRα mRNA expression correlated with the concentration of liver fat, which was interpreted as having a lipogenic effect on this organ that requires consideration, similar to an observation that has been previously made by others [38].

Ruiz-Canizales et al. [61] used freeze-dried mango (*Mangifera indica*) pulp, which was administered whole or as only its isolated fiber or phenolics (0.1% *w*/*w*), as part of a high-cholesterol/sodium cholate diet. An increased mRNA expression of LXRα was found for the fiber and phenolics groups, but not for the group that consumed whole mango, suggesting the targeted effect of isolated compounds. The effects on LXRα were interpreted as part of a mechanism of action that exerted hepatoprotection against the toxic effects of cholesterol/sodium cholate. It is interesting to note that the effects of pecan [60] and mango phenolics [61] on LXRα were significant, and could not be replicated by their respective sources when administered whole. This suggests that phenolic compounds are, at least in part, responsible for the bioactivities documented by pecan and mango consumption, and that their effects are retained and/or increased when extracted and administered in concentration.

Olive oil consumption has been associated with various health effects, partly due to its phenolic content. Farràs et al. [62] performed a randomized, double-blind, crossover trial, in which 22 hypercholesterolemic adults (total cholesterol > 200 mg/dL) consumed 25 mL/day of olive oil enriched with olive oil phenolics (500 mg/kg), or olive oil enriched with olive oil (250 mg/kg) and thyme phenolics (250 mg/kg). After a three-week period, the mRNA expression of LXRβ and RXRα (as well as other cholesterol-efflux-related genes) was increased by the olive- and thyme-enriched oil in the peripheral blood mononuclear cells. Their findings allowed the authors to propose that their treatments simultaneously acted on the PPAR and LXR pathways, bioactivities that resulted in a significant increase in cholesterol efflux. This is congruent with a previous experiment, in which the same olive oil treatments were predicted (ingenuity pathway analysis) to have a significant impact on the LXR/RXR pathway [63], as well as with various other in silico, in vitro, and in vivo models discussed in the present work. The actual phenolic profile of the oils is not reported; however, hydroxytyrosol is a well-known phenolic that is characteristic of olive oil, and has been associated with multiple bioactivities. This compound was studied by Franceschelli et al. [64], who administered it (25, 50, or 100 μM for 24 h) to foam cells, and determined a significant cholesterol clearance effect. This was associated with the LXR and PPAR pathways, according to increased LXRα mRNA and protein expression, as well as increased PPARγ mRNA expression in response to this phenolic. Taken together, these studies support the role of olive oil phenolics in general and hydroxytyrosol in particular, as modulators of cholesterol metabolism, with a mechanism of action based on targeting LXR and other related nuclear receptors.

Byproducts from fruit processing, such as peels and seeds, are commonly discarded after the edible pulp is obtained for various purposes; however, these vegetable tissues have also been shown to contain significant concentrations of bioactive compounds. Tan et al. [65] extracted phenolics from longan fruit (*Dimocarpus longan* Lour.) pericarps and seeds, and administered this extract (0.2%, 12 weeks) to C57BL/6J mice that were fed an HFD (60% energy from fat). Anti-obesity (reduced weight gain) and hypolipidemic (decreased serum and hepatic lipids) effects suggested protection against the metabolic alterations commonly exerted by consuming an HFD. Increased mRNA and protein expression of LXRα and PPARα were documented, as well as some of their target genes, which may be responsible for the observed protection. Phlorizin, proanthocyanidin A2, and gallic acid were the main compounds present in the extract, as well as eight less abundant ones. It could be argued that the most concentrated molecules may be responsible for the observed effects; however, minor compounds can also contribute, in addition to potential synergies between them, as previously mentioned for other phenolics. This work thus reveals the potential of byproducts being used as sources of bioactive phenolics, capable of exerting significant bioactivities on the LXR pathway.

According to the previously mentioned in silico, in vitro, and in vivo data, it is apparent that different classes of phenolic compounds are able to exert health effects by targeting nuclear receptors. This was documented when the compounds were administered by themselves, in synergy with other molecules, as part of an extract, or by some of their metabolites. The data show that their bioactivities can be observed at the mRNA expression and/or protein levels and they can be cell- and dose-dependent, and specificity for some isoforms over others is also shown. A summary of the studies discussed in this section is provided in Table 1.

## 6. Perspectives

Multiple bioactivities of phenolic compounds can be found in the literature, according to studies that demonstrate health-promoting results across various cells and organs in response to consuming them. Although most experiments propose a given mechanism by which a certain documented effect is exerted, it is possible that at least some effects are due to modulating nuclear receptors. Regulating LXR and RXR at the mRNA and/or protein levels is a potential mechanism that may underlie at least some of the documented effects of phenolic compounds. RXR in particular stands out, since it is not involved in one specific pathway, but is instead capable of participating in numerous ones. As mentioned in the present work, phenolic compounds can modulate either RXR, one of its obligate heterodimer-forming partners, or both, which results in such varied responses like altering lipid, bile, or xenobiotic metabolism, among others. In other words, if a phenolic compound is able to modulate RXR, it may consequently be able to have a significant impact on multiple tissues and metabolic processes at once, since targeting RXR may directly or indirectly alter its interactions with other transcription factors, resulting in a variety of different bioactivities. The fact that many wide-ranging processes under the partial control of RXR may be regulated by phenolic compounds may help to explain why they are so bioactive in vivo.

The evidence presented shows promise, but also reveals some noteworthy caveats. For example, the effects of some compounds tend to be studied for RXR or one of its heterodimer-forming partners (such as LXR), but not always for both, which suggests that some information may be missing by not considering one-half of the obligate dimer. In fact, less attention is given to RXR, even while it is such a significant participant in the regulation of numerous metabolic processes.

How or even whether a given phenolic interacts directly with a nuclear receptor is seldom reported. This data could be relevant to determine why some compounds show marked specificity toward one specific isoform, while having minimal or no effect on another. Determining which domains and/or specific residues of the nuclear receptor participate in these potential interactions may reveal key information, which could be used to further clarify how phenolic compounds exert their bioactivities and to develop additional—and perhaps even more potent—derivatives.

When a complex mixture is administered, it can be impossible to determine which molecule or molecules are responsible for the observed effect. Although mixtures remain significant given they are closer to how a compound would be expected to be found when consumed as part of a normal diet (i.e., as part of a mixture and not in isolation), some information cannot be precisely known under such circumstances.

Regarding the sources of currently available information, most has been gathered from in vitro and animal models, since it is often impossible to obtain it from humans. Although these models are certainly invaluable, they are still only approximations of what could be happening in humans. Information from humans is therefore required to verify in silico predictions and results from in vitro and in vivo experiments.

Finally, a normal diet is highly variable according to age, sex, geographical location, time of year, personal preferences, health status, among other things, which, due to the many variables involved, means determining how a given dietary compound may interact in vivo is further complicated. In the case of phenolics, their diverse chemical features like their monomeric/polymeric structure, glycosylation patterns, and the presence and position of substituents adds another layer of complexity to this analysis. Moreover, the concentration of phenolics may differ across a plant’s tissues due to normal diurnal and seasonal variations, climate, geography, and other factors; additional variation is also likely to occur due to changes during harvest, transport, storage, and meal preparation. Such differences may be reflected in the health effects exerted on the consumer.

Based on this, it is apparent that there is a lot of work required to fully understand how dietary bioactives like phenolic compounds modulate health at the molecular and gene expression levels. Additional in silico and in vitro experiments are necessary, but human-derived data should also be considered whenever possible. Continued investigation into this topic is likely to benefit healthy individuals in order to preserve their health, as well as patients that are already suffering from some diet-related non-communicable diseases that are highly prevalent throughout the modern world, including obesity, diabetes, certain types of cancer, among others.

## 7. Conclusions

Nuclear receptors are multi-functional ligand-activated signal transducers, whose role across multiple cellular functions makes them ideal targets for health-promoting molecules. Phenolic compounds have been shown to exert multiple bioactivities, with the recent literature suggesting that at least some of them are exerted via targeted effects on liver X receptor (LXR) and/or retinoid X receptor (RXR). If LXR is targeted, various lipid-related processes may be modulated, according to its significant effect on them. A similar response may also occur if RXR is instead targeted, since it can dimerize with LXR and thus act on the same genes, although it can also have an impact on multiple other processes. More precise evidence suggests that the phenolic compound–nuclear receptor interactions can also be selective for a particular isoform since one of them may show significant responses to a phenolic compound, while another may have a weak response or none at all. The interactions and responses may also be cell-specific and/or dose-dependent. Additional experiments are required to conclusively determine how a particular phenolic compound can interact with LXR or RXR, and how these interactions result in the observed effects. Continued study of foods rich in phenolic compounds and their effects on health is still warranted.

## Figures and Tables

**Table 1:** Summary of the effects of phenolic compounds on liver X receptor (LXR) or retinoid X receptor (RXR) discussed in the main text.

Phenolic Treatment	Bioactivity Documented	Effect on LXR or RXR	Reference
Quercetin (5, 10, or 15 μM)	Increased mRNA and protein of cholesterol efflux genes in vitro	Increased LXRα mRNA and protein	[29]
Quercetin (10, 50, or 250 μM)	Potential immunomodulation and anti-cancer effects in vitro	Cell-dependent increase or decrease in LXRα and RXRα mRNA	[30]
Resveratrol (0.8–500 mg/kg body weight oral gavage or 0.1 or 0.5% *w*/*w* dietary supplementation)	Improved lipid profile and fecal sterol excretion in vivo, increased expression of cholesterol transporters in vitro	Selective effect on LXRα, but not on LXRβ	[31]
Resveratrol (1, 25, 125, or 625 μM)	Potential antiproliferative effects in vitro	Cell-dependent increase or decrease in RXRα mRNA	[32]
Resveratrol (10^−5^ M) with or without vitamin D_3_ (10^−8^–10^−10^ M)	Increased vitamin-D-related transcription in vitro	Formation and/or stabilization of VDR/RXR heterodimer	[34]
Resveratrol (40 or 400 mg/kg)	Altered reverse cholesterol transport in vivo independently of resveratrol	No effect on LXR signaling	[35]
Resveratrol (15 mg/kg/day) and a synthetic derivative (SY-102, 45 mg/kg/day)	Hepatoprotection in vivo	Modulated the AMPK/LXR/SREBP pathway	[36]
Oxyresveratrol (10 or 30 mg/kg)	Inhibit hepatic lipogenesis and consequent hepatoprotection in vivo	Modulated the LXRα/SREBP-1c pathway	[37]
5-caffeoylquinic acid (20 or 90 mg/kg)	Hepatoprotection in vivo	Normalized LXRα mRNA, increased RXRα mRNA	[38]
Chlorogenic acid (300 or 600 mg/kg diet)	Improvements to the lipid profile; antioxidant and anti-inflammatory effects in vivo	Increased LXRα mRNA, no effects on RXRα	[39]
Chlorogenic acid (0.1 or 0.2%) and caffeine (0.02 or 0.04%) combined	Decreased macrophage and hepatocyte lipid content in vitro	Decreased LXRα mRNA when combined with caffeine; no effect by itself	[40]
3-caffeoyl, 4-dihydrocaffeoylquinic acid (1–30 μM)	Hepatoprotection in vitro	Did not normalize LXRα mRNA expression	[41]
Cyanidin (5, 10, 50, or 100 μM)	Decreased macrophage and hepatocyte lipid content in vitro	Increased LXRα and LXRβ mRNA, increased expression of LXRα-regulated genes but not LXRβ, higher affinity for LXRα	[42]
Cyanidin-3-O-β-glucoside chloride or cyanidin chloride (50 μM)	Increased cholesterol efflux and anti-inflammatory effects in vitro	Modulated the LXRα pathway	[43]
Quercetin and ferulic acid (1 μM of each or both)	Modulated lipid metabolism in vitro	Increased mRNA of genes controlled by the PPARα/RXRα pathway	[44]
Galangin, quercetin, apigenin, and naringenin (1, 10, or 100 μM individually administered)	Various phenolic–protein interactions determined in silico, potential lipid-modulating effects in vitro	Quercetin increased LXRα and LXRβ mRNA, apigenin increased LXRβ mRNA, no effect of galangin and naringenin	[46]
Phenolic-rich Chinese herb extract (250 mg/kg diet in vivo, 200 μg/mL in vitro); phenolic acids (caffeic acid and vanillin, 150 μg/mL in vitro); or flavonoids (daidzein and quercetin-d-glucoside, 50 μg/mL in vitro)	Potential effects on bile and xenobiotic metabolism; possible synergy between phenolics	Phenolic acids or phenolic acids and flavonoids increased RXRα mRNA	[47]
Curcumin (40 mg/kg)	Normalized gene expression of cancer stem cells in vitro	Normalized RXRα mRNA expression	[48]
Curcumin (50 or 100 mg/kg)	Improved cognitive parameters of rats with cerebral hypoperfusion	Modulated LXRβ and RXRα mRNA expression	[49]
Curcumin (50 or 100 mg/kg)	Hepatoprotection in vivo	Modulated the Nrf2/FXR/LXR pathway	[50]
Curcumin and halogenated derivatives of curcumin (3.75 × 10^−5^ M), and resveratrol (2.8 × 10^−5^ M)	Synergy between resveratrol and curcumin and its halogenated derivatives in vitro	Modulated RXR/VDR signaling	[51]
Phenolic-rich extract of *Prumnopitys andina* (1, 10, 50, 100, and 200 μg/mL)	Potential lipid modulation and anti-inflammatory effects in vitro	Increased mRNA expression of LXRα and LXRβ	[52]
Cornelian cherry (*Cornus mas* L.) extract (10 or 50 mg/kg bw)	Improved lipid profile in vivo	Increased LXRα protein	[53]
Non-anthocyanin cherry (*Prunus avium* L.) phenolics (629 ± 39 and 130 ± 3.9 mg gallic acid equivalents/kg diet)	Hepatoprotection and anti-inflammatory effects in vivo	Normalized LXRβ mRNA	[54]
Loquat (*Eriobotrya japonica* (Thunb.) Lindl.) fruit peel extract (100 or 200 mg/kg/day)	Improved lipid profile in vivo	Potential in silico interactions between phenolics and some of their metabolites with LXRα, RXRα, RXRβ, and RXRγ	[55]
Cocoa flavonols (10 μM)	Potential modulation of apolipoprotein A1 in vitro	Binding of RXRα to site A of the APOA1 promoter	[56]
Green tea catechins (0.24%)	Anti-tumor effects in vivo	Increased mRNA expression of RXRα, β, and γ	[57]
Epigallocatechin-3-gallate (50, 100, or 150 μM)	Potential anti-tumor effects in vitro	Restored epigenetically silenced RXRα expression	[58]
Apple polyphenol extract (125 or 500 mg/kg/day)	Hepatic and intestinal protection in vivo	Increased ileal LXRα mRNA expression	[59]
Isolated pecan (*Carya illinoinensis*) phenolics (0.1% *w*/*w*)	Anti-atherogenic effects in vivo	Increased LXRα mRNA expression	[60]
Isolated mango (*Mangifera indica*) phenolics (0.1% *w*/*w*)	Hepatoprotection against high-cholesterol/sodium cholate diets in vivo	Increased LXRα mRNA expression	[61]
Olive oil enriched with olive oil and thyme phenolics (500 mg/kg of phenolics, 25 mL/day)	Potential cholesterol clearance from peripheral blood mononuclear cells in hypercholesterolemic adults	Increased LXRβ and RXRα mRNA expression	[62]
	Changes to the HDL-associated proteome of hypercholesterolemic adults	Predicted effects on LXR/RXR	[63]
Hydroxytyrosol (25, 50, or 100 μM)	Increased cholesterol clearance from foam cells in vitro	Increased LXRα mRNA and protein expression	[64]
Phenolic-rich extract from longan (*Dimocarpus longan* Lour.) byproducts (0.2%)	Anti-obesity and hypolipidemic effects in vivo	Increased LXRα mRNA and protein expression	[65]

## Data Availability

No new data were created or analyzed in this study. Data sharing is not applicable to this article.
